# Role of MRI in the Diagnosis of Large Right Adrenal Abscess

**DOI:** 10.7759/cureus.10986

**Published:** 2020-10-16

**Authors:** Matthew Stankard, Dheeraj Gopireddy, Chandana Lall

**Affiliations:** 1 Radiology, Florida Atlantic University (FAU) College of Medicine, Boca Raton, USA; 2 Radiology, University of Florida College of Medicine, Jacksonville, USA; 3 Abdominal Imaging, University of Florida College of Medicine, Jacksonville, USA

**Keywords:** role of mri, abdominal pseudocyst, adrenal mass, adrenocortical carcinoma, adrenal abscess, body mri, adrenal hemorrhage

## Abstract

Adrenal abscesses are extremely rare occurrences with only scattered case reports reported in the literature. Owing to their rarity, they are not usually considered within the typical differential for cystic adrenal masses discovered on computed tomography (CT) or magnetic resonance imaging (MRI). Accurate and timely diagnosis of these lesions is critical to exclude malignancy and ensure appropriate management. In this case report, we describe a post-traumatic case of adrenal abscess associated with Staphylococcus aureus bacteremia and its differentiation from cystic adrenal masses. Specific emphasis is placed on the imaging features seen on CT and MRI and how these features can be utilized to differentiate it from other adrenal pathologies such as adrenal pseudocyst or cystic adrenocortical neoplasm.

## Introduction

Adrenal lesions cover a broad spectrum of benign to malignant entities and are most often detected incidentally on computed tomography (CT) and magnetic resonance imaging (MRI). Once identified, a specific evaluation must be performed to exclude malignancy and determine functional status. Primary malignancy is an uncommon cause of incidentally discovered adrenal masses, with the frequency of primary adrenal carcinoma estimated to be between 1.9% and 4.7%, and the frequency of metastasis estimated to be 0.7% to 2.3% [1]. In addition to the determination of benign and malignant adenomas, cystic lesions, such as endothelial cysts, pseudocysts, epithelial cysts, and parasitic cysts, can present similarly on imaging to adrenal abscess and must be accurately excluded from the diagnosis.

Adrenal abscesses are often a consequence of hematological seeding of the adrenal gland following hemorrhage. These rare lesions are most commonly encountered in the setting of disseminated infection and have been reported in the literature to occur in conjunction with both Nocardia spp. and Streptococcus pneumonia [2-5]. Though often caused secondarily to fatal bacterial infection, non-infectious causes of adrenal hemorrhage include postoperative state, use of anticoagulants, burns, trauma, tumor metastasis, and cardiovascular collapse [6].

This article presents the case of a large cystic adrenal mass found in the setting of Staphylococcus aureus bacteremia and illustrates the diagnostic challenges in distinguishing between adrenal abscess, adrenal pseudocyst, and necrotic adrenocortical carcinoma on CT and MRI [2]. After extensive imaging analysis, the patient was sent to interventional radiology (IR) for percutaneous abscess drainage where the diagnosis was confirmed with an aspirate of infectious fluid, resolution of symptoms, and follow-up of microbiological analysis.

## Case presentation

**Figure 1:**
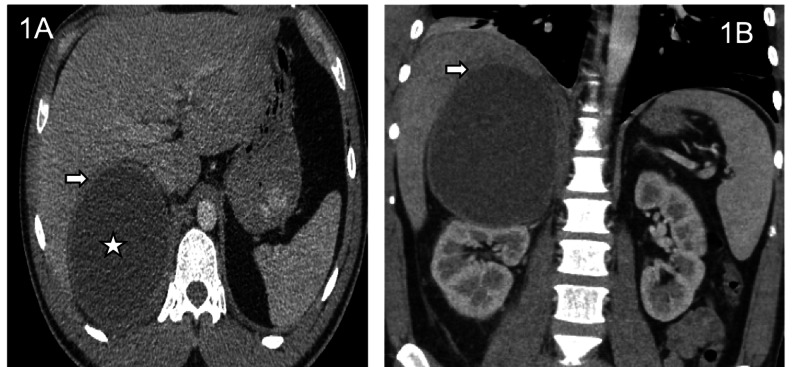
Axial and Coronal Post-Contrast CT Venous Phase Axial (1A) and coronal (1B) post-contrast CT venous phase demonstrate a large low attenuating cystic mass in the right perinephric space in the location of the right adrenal gland (star). Notice the mass effect on the right hepatic lobe (arrows). CT: computed tomography

A 32-year-old male with no past medical history presented to the emergency department with the chief complaint of a two-week history of fevers, right upper quadrant (RUQ) abdominal pain, and a 13-pound weight loss secondary to loss of appetite. He worked as a landscaper and denied intravenous drug use though he did self-administer testosterone injections. He reported a motorcycle accident five months ago where he injured his right side and thought he may have broken two ribs on his right. In the ED, he was febrile at 100.6 degrees but hemodynamically stable. On labs, he had a white blood cell (WBC) count of 12.24, comprehensive metabolic panel (CMP) was within normal limits, liver function panel was within normal limits, and urinalysis was normal. The human immunodeficiency virus (HIV) test was negative. Initial CT abdomen and pelvis with contrast revealed a large right suprarenal 10 x 10 x 14 cm rim-enhancing cystic mass with areas of irregular wall thickening along the inferior margin and infiltration of the surrounding fat (Figures 1A-1B).

There was a loss of distinct fat planes between the mass, the right hepatic lobe, and the superior portion of the right adrenal gland, therefore, a neoplastic process of hepatic or adrenal origin was unable to be excluded. Follow-up with an MRI abdomen with and without contrast was recommended.

He was started on intravenous (IV) vancomycin and piperacillin/tazobactam. Blood cultures returned one of two positive for gram-positive cocci in clusters suggestive of staphylococcus aureus. This was initially thought to represent contamination, though this was later confirmed with abdominal lesion aspirate culture. IV antibiotics were transitioned to cefazolin and metronidazole.

**Figure 2:**
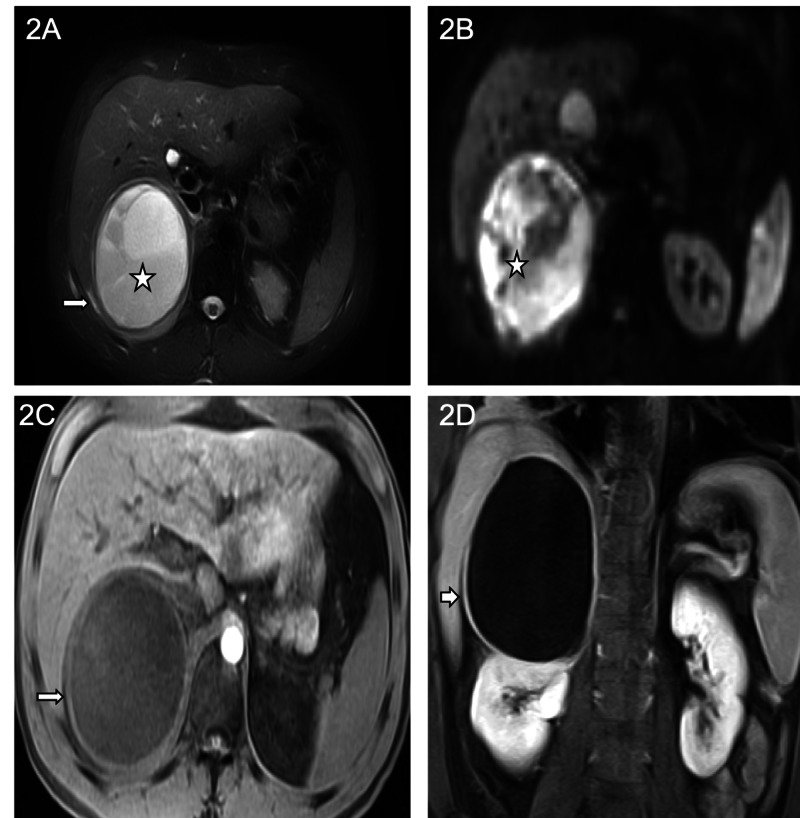
MRI Abdomen Axial T2 fat saturation, pre-contrast T1 weighted (T1 W), post-contrast T1W, and diffusion-weighted imaging (DWI) images, respectively, demonstrate a large T2 bright cystic mass (star) with a rim of edema surrounding the mass (arrow) (2a). On the DWI B500 (2b), there is marked restricted diffusion (star) in the entire cystic mass supporting an abscess. On pre and post-contrast T1W images (2c, 2d), there is a smooth enhancement of the wall (arrow). Notice there is no nodularity of the wall.

Two days later, MRI results showed a large cystic lesion that was T2-hyperintense and T1-hypointense with mild internal layering material, measuring approximately 9.7 x 10.0 x 13.6 cm (anteroposterior (AP) x transverse (TRV) x craniocaudal (CC)). There was a smooth enhancement of the cyst wall or nodularity of the wall after the administration of contrast. Internal contents demonstrated signal changes consistent with proteinaceous blood products. T2 bright edema was identified surrounding the lesion within the perinephric space with a significant mass effect seen along the posterior segment of the right hepatic lobe and upper pole of the right kidney (Figures 2A-2D).

IR was consulted for ultrasound-guided drainage of the right retroperitoneal abscess collection with the immediate aspiration of serosanguinous fluid. Acid-fast bacilli culture of the abdominal drainage was negative, fungal culture was negative, and bacterial culture positive for Staphylococcus aureus with many neutrophils.

The patient continued to improve clinically and was discharged on antibiotics.

## Discussion

In the present case, the large adrenal cystic mass seen on CT and MRI, in combination with the patients' symptoms, support a diagnosis of adrenal abscess secondary to trauma-induced adrenal hemorrhage.

There is a relative lack of literature on large adrenal abscesses owing to the uncommon nature of these lesions. Adrenal abscesses have been reported in the literature to occur secondary to a placental infection, infected adrenal hemorrhage, immunocompromised states, and in association with pheochromocytomas [3-5,7]. Adrenal abscesses can be easily misdiagnosed owing to the uncommon nature of these findings and the diagnostic difficulty in distinguishing between adrenal cystic pathologies such as pseudocysts with or without hemorrhage, and other necrotic adrenal tumors.

In cases where malignancy is considered, certain imaging characteristics are more suggestive of adrenocortical carcinoma or metastasis. These include the irregular shape of the wall, heterogeneous density due to central areas of necrosis, diameter > 4 cm, presence of calcifications, delayed contrast washout, and high CT attenuation > 20 HU on unenhanced CT [8]. Benign lesions, on the other hand, tend to be smaller, with diameter < 4 cm, are more round and homogenous with smooth contours, have low unenhanced CT attenuation, and rapid contrast washout [9-10].

MRI has been shown to be an effective diagnostic tool in the evaluation and characterization of adrenal tumors, with a reported sensitivity of 89% and a specificity of 99% [11]. On MRI, benign adenomas often show isointensity with liver on T1 and T2-weighted MRI sequences while an adrenocortical carcinoma displays isointensity or mild hypointensity with liver on T1-weighted MRI and high to intermediate signal intensity on T2-weighted MRI [8]. Adrenocortical carcinoma is typically heterogeneous in signal intensity due to the presence of hemorrhage or necrosis [8]. The presence of heterogeneity seen in typical adrenocortical carcinoma makes diagnosis particularly difficult, as necrosis can easily mimic the internal complicated components seen in abscesses and pseudocysts.

Adrenal cysts are rare and account for approximately 5% of incidentally discovered cystic lesions [12]. Most are benign, though approximately 7% of lesions are malignant [13]. Adrenal cysts are classified into four different types: endothelial cysts (45%), pseudocysts (39%), epithelial cysts (9%), and parasitic lesions (7%) [14].

Endothelial cysts typically present as low-density masses with smooth borders and thin walls. On MRI, they are homogeneously hypointense on T1-weighted images and hyperintense on T2-weighted images with no internal enhancement [15].

Adrenal pseudocysts on the other hand are usually caused by an episode of adrenal hemorrhage or infarct and are composed of fibrous tissue and characteristically lack endothelial lining. On CT, pseudocysts are round masses with fluid density and often display complicated internal components such as blood, septa, or soft tissue [15]. They contain calcifications more frequently than endothelial cysts (20% vs. 9.5%) [15-16]. These complicated internal cystic components are best evaluated by MRI, especially in cases of intracystic hemorrhage, which shows hyperintensity on T1- and T2-weighted images [15]. Due to the presence of these intracystic components, there have been multiple case reports of adrenal pseudocyst mimicking neoplastic processes [16-18]. In our current case, marked uniform restricted diffusion and surrounding edema in the perinephric space favored the diagnosis of an infected pseudocyst.

## Conclusions

This case demonstrates the diagnostic difficulty in differentiating adrenal abscess, adrenal pseudocyst, and necrotic adrenocortical carcinoma from one other. When considering these diagnoses, evaluation with MRI is critical in order to best characterize the internal components of the lesion. In addition, special attention should be paid to clinical presentation. Adrenal abscess should be especially considered when patients present with signs of infection such as the two-week history of fevers seen in this patient. MRI imaging with superior soft-tissue resolution can aid in a more accurate diagnosis of adrenal abscess and differentiate between necrotic adrenocortical carcinoma or adrenal pseudocyst.

## References

[1] Cawood TJ, Hunt PJ, O’Shea D, Cole D, Soule S (2009). Recommended evaluation of adrenal incidentalomas is costly, has high false-positive rates and confers a risk of fatal cancer that is similar to the risk of the adrenal lesion becoming malignant; time for a rethink?. Eur J Endocrinol.

[2] Midiri M, Finazzo M, Bartolotta TV, De Maria M (1998). Nocardial adrenal abscess: CT and MR findings. Eur Radiol.

[3] Chong YL, Green JA, Toh KL, Tan JK (2004). Laparoscopic drainage of nocardial adrenal abscess in an HIV positive patient. Int J Urol.

[4] Jackson C, McCullar B, Joglekar K, Seth A, Pokharna H (2017). Disseminated Nocardia farcinica pneumonia with left adrenal gland abscess. Cureus.

[5] Urrutia A, Santesmases J, Benítez RM, Areal J (2010). Adrenal gland abscess due to Streptococcus pneumoniae. J Infect.

[6] Xarli VP, Steele AA, Davis PJ, Buescher ES, Rios CN, Garcia-Bunuel R (1978). Adrenal hemorrhage in the adult. Medicine.

[7] Inoue R, Hisasue S-I, Kunishima Y, Masumori N, Itoh N, Tsukamoto T (2007). Pheochromocytoma with abscess. Int J Urol.

[8] Bharwani N, Rockall AG, Sahdev A, Gueorguiev M, Drake W, Grossman AB, Reznek RH (2011). Adrenocortical carcinoma: the range of appearances on CT and MRI. Am J Roentgenol.

[9] Jordan E, Poder L, Courtier J, Sai V, Jung A, Coakley F V (2012 199). Imaging of nontraumatic adrenal hemorrhage. Am J Roentgenol.

[10] Kawashima A, Sandler CM, Fishman EK (1998). Spectrum of CT findings in nonmalignant disease of the adrenal gland. Radiographics.

[11] Hönigschnabl S, Gallo S, Niederle B (2002). How accurate is MR imaging in characterisation of adrenal masses: update of a long-term study. Eur J Radiol.

[12] Masumori N, Adachi H, Noda Y, Tsukamoto T (1998). Detection of adrenal and retroperitoneal masses in a general health examination system. Urology.

[13] Neri LM, Nance FC (2020). Management of adrenal cysts. Am Surg.

[14] Foster DG (2020). Adrenal cysts: review of literature and report of case. Arch Surg.

[15] Guo YK, Yang ZG, Li Y, Deng Y-P, Ma E-S, Min P-Q, Zhang X-C (2007). Uncommon adrenal masses: CT and MRI features with histopathologic correlation. Eur J Radiol.

[16] Bovio S, Porpiglia F, Bollito E (2007). Adrenal pseudocyst mimiking cancer: a case report. J Endocrinol Invest.

[17] Isono M, Ito K, Seguchi K (2017). A case of hemorrhagic adrenal pseudocyst mimicking solid tumor. Am J Case Rep.

[18] Passoni S, Regusci L, Peloni G, Brenna M, Fasolini F (2013). A giant adrenal pseudocyst mimicking an adrenal cancer: case report and review of the literature. Urol Int.

